# Adsorption of Silicon-Containing Dendrimers: Effects of Chemical Composition, Structure, and Generation Number

**DOI:** 10.3390/polym13040552

**Published:** 2021-02-13

**Authors:** Andrey O. Kurbatov, Nikolay K. Balabaev, Mikhail A. Mazo, Elena Yu. Kramarenko

**Affiliations:** 1 Faculty of Physics, Lomonosov Moscow State University, 119991 Moscow, Russia; kurbatov@polly.phys.msu.ru; 2 A. N. Nesmeyanov Institute of Organoelement Compounds RAS, 119991 Moscow, Russia; 3 Institute of Mathematical Problems of Biology, Keldysh Institute of Applied Mathematics RAS, Pushchino, 142290 Moscow, Russia; balabaevnk@gmail.com; 4 Semenov Institute of Chemical Physics RAS, 119991 Moscow, Russia; mikhail.mazo1@gmail.com

**Keywords:** dendrimer, polybutylcarbosilane dendrimer, siloxane dendrimer, adsorption, molecular dynamics

## Abstract

We studied the conformational behavior of silicon-containing dendrimers during their adsorption onto a flat impenetrable surface by molecular dynamics (MD) simulations. Four homologous series of dendrimers from the 4th up to the 7th generations were modeled, namely, two types of carbosilane dendrimers differing by the functionality of the core Si atom and two types of siloxane dendrimers with different lengths of the spacers. Comparative analysis of the fractions of adsorbed atoms belonging to various structural layers within dendrimers as well as density profiles allowed us to elucidate not only some general trends but also the effects determined by dendrimer specificity. In particular, it was found that in contrast to the carbosilane dendrimers interacting with the adsorbing surface mainly by their peripheral layers, the siloxane dendrimers with the longer –O–Si(CH_3_)_2_–O spacers expose atoms from their interior to the surface spreading out on it. These findings are important for the design of functional materials on the basis of silicon-containing dendrimers.

## 1. Introduction

Dendrimers are hyperbranched polymer molecules with a regular structure. They consist of a branching core atom or an atomic group with at least two functionalities, spacers of a fixed length, branching points that represent an atom or atomic group with at least three functionalities, and terminal groups. The potential for dendrimer synthesis was theoretically predicted in the 1950s [1], but the first dendrimers were obtained only in the late 1970s [2], and there has been a continuing interest in dendrimers in the last years. Nowadays, the main application of dendrimers is in medicine [3] for the encapsulation of various medicinal agents [4,5]. Furthermore, dendrimer molecules have a large number of end groups that can be modified according to needs [6,7,8,9]. Medicinal or other agents, labels to track the movement of molecular complexes throughout the body, molecular groups that increase solubility, and other modifiers can be attached to them. In addition, dendrimers are used to create biosensors [10,11,12,13]. Since they have the maximum number of end groups at a fixed molecular weight, dendrimers turn out to be more effective for the design of structured functional surfaces than linear polymers.

One of the first works on the dendrimer adsorption onto a solid impenetrable planar surface was focused on conformation changes of dendrimer molecules depending on the strength of adsorption [14]. It was shown by computer simulations that there are several adsorption modes differing in the dynamics and conformations of dendrimers (spherical or more oblate), and the transitions between these modes depend not only on the adsorption strength but also on the dendrimer generation. Subsequently, most of the research on the adsorption of dendrimers was carried out by experimental methods and mainly for poly(amidoamine) (PAMAM) and poly(propylene-imine) (PPI) dendrimers with different functional groups. Basically, the main driving force for the adsorption of these dendrimers was the electrostatic interaction with the surface [15,16,17,18,19,20,21,22].

The general trends in the adsorption behavior of dendrimers that can be drawn from experimental studies are following. First, the behavior of dendrimers near a surface is mainly determined by mechanisms of interaction with the surface [14,15,16,17,18,19,20,21,22]. Second, there are certain differences in adsorption behavior between dendrimers of low and high generations, which is due to an increase in the size of the molecules and conformational restrictions that manifest themselves during the adsorption process [14,15,16,17,18,19,20,21,22]. Therefore, if one approaches this issue from a theoretical point of view, or via computer simulations, it is quite acceptable to neglect all effects of external conditions such as, for instance, the solvent quality, in comparison with the type of potential describing dendrimer interactions with the surface.

Up to now, computer simulations of the dendrimer adsorption were mainly focused on (i) differences in adsorption depending on which particular atoms are attracted to the surface and on (ii) effects of the nature of attractive interactions, i.e., either Coulomb or hydrophobic [23,24]. In the case of Coulomb attraction, the repulsion between similarly charged monomer units of the adsorbing dendrimer affects considerably its final conformation, making it more stretched, regardless of which particular structural units of the dendrimer are attracted to the surface [23]. In contrast, the hydrophobic mechanism of attraction leads to a denser structure of adsorbed dendrimers [24]. If both adsorption mechanisms are involved, then the final conformation is closer to that determined by electrostatics [24]. It was also shown that during adsorption, dendrimers form a two-layered structure normal to the surface consisting of an adsorbed dense layer and a looser remaining part [24].

There are certain differences between the adsorption behavior of low- and high-generation dendrimers; it depends on geometric parameters of dendrimers such as the length of the spacers, the functionality of the branching points, and the dendrimer core [25]. It can be argued that for fixed geometric parameters of the dendrimer that the dendrimer experiences a conformational transition in which it spreads out along the surface with an increase in the adsorption force. This step-like transition from the weak to the strong adsorption regime occurs through overcoming the potential barrier caused by elastic forces and steric effects. If the adsorption strength is fixed, then a transition in the adsorption behavior between low and high generations occurs.

Although general trends in the adsorption behavior of dendrimers have been identified, it is worth noting that conclusions have mainly been drawn from an analysis of the behavior of PAMAM and PPI dendrimers [15,16,17,18,19,20,21,22] or general model structures [14,23,24,25]. That is, mainly the case of strong adsorption due to the Coulomb interaction was studied experimentally, while theoretical works, on the contrary, did not sufficiently take into account the potential features of the chemical composition of dendrimers.

From this point of view, silicon-containing dendrimers, in particular, carbosilane and siloxane ones, containing no atomic groups with any specific interactions such as hydrogen bonding or electrostatic interactions, are of particular interest. They can serve as good model systems to study the effects of a regularly branched structure and elucidate some features of dendrimer behavior during adsorption due to their tree-like architecture.

In the past few years, an interest in the study of these dendrimers in bulk has been increasing [26,27,28,29,30,31,32,33,34,35,36,37,38]. Research was carried out both by experimental methods and by computer simulations. The homologous series of carbosilane dendrimers with a core functionality of four and a branch point functionality of three (4-3 dendrimers) is the most studied, including dendrimers up to the 8th generation. The most significant phenomenon detected is an unprecedented jump in the viscosity of the melt with increasing generation or, in fact, the molecular weight of the molecules [26]. It has been found that the melts of dendrimers up to the fifth generation are liquids, while starting from the sixth generation, dendrimer melts demonstrate a solid-like behavior. Currently, there is no exact explanation of such critical behavior with generation number; however, it is clear that the regular tree-like structure of dendrimers is one of the major factors responsible for this liquid–solid transition, which is localized in a specific generation range. A critical behavior is also typical for dendrimers during their interaction with the surface, as was mentioned above. Thus, a more detailed study of carbosilane dendrimers near the adsorbing surface, which would take into account the atomic structure of these macromolecules, is important for a better understanding of the general influence of the generation number on their conformational behavior. Dendrimers of the siloxane families, synthesized about 30 years ago [37,38], have just begun to be studied [29,30], and their modeling is very interesting and has a predictive value. Furthermore, recently, some works appeared on the synthesis and study of physical properties of hybrid dendrimers consisting of segments of various chemical natures [39,40], which have strong potential for the creation of membrane materials. In this regard, computer simulations of the adsorption of dendrimers at the atomistic level may provide some insight into how the individual elements of different chemical nature work for the targeted synthesis of materials with desired properties.

The aim of this work is to identify specific features of the adsorption behavior of silicon-containing dendrimers, including carbosilanes, due to their structure and chemical composition. The urgent issue is to verify possible deviations from the predictions of coarse-grained models and theoretical estimations; the latter are basically relevant for systems with a large number of monomer units, tending to the limiting case of infinity. Our first goal is to answer the question of how acceptable the use of coarse-grained models is in comparison with atomistic models; our second is to find some features of the adsorption of dendrimers, depending on the chemical nature of atomic bonds, as well as the details of the structure of dendrimers; finally, our third is to find some clues about the criticality of dendrimer behavior as the generation number increases.

In the next section, the structure of silicon-containing dendrimers under study as well as the method of simulations are introduced. In Section 3, we first discuss the range of reasonable values of the adsorption parameter, which covers the regimes of both weak and strong adsorptions without any considerable stress in the dendrimer structure. Then, a comparative analysis of the number of contacts of dendrimer monomers with the surface (total number and the number per structural layer) is performed depending on the adsorption energy, dendrimer specificity, and generation number. Finally, the conformational changes reflected in density profiles and shape transformations are studied for the four homologous series of dendrimers at various adsorption energies. The main results are summarized in the Conclusion section.

## 2. Research Objects and Method of Simulations

**Figure 1:**
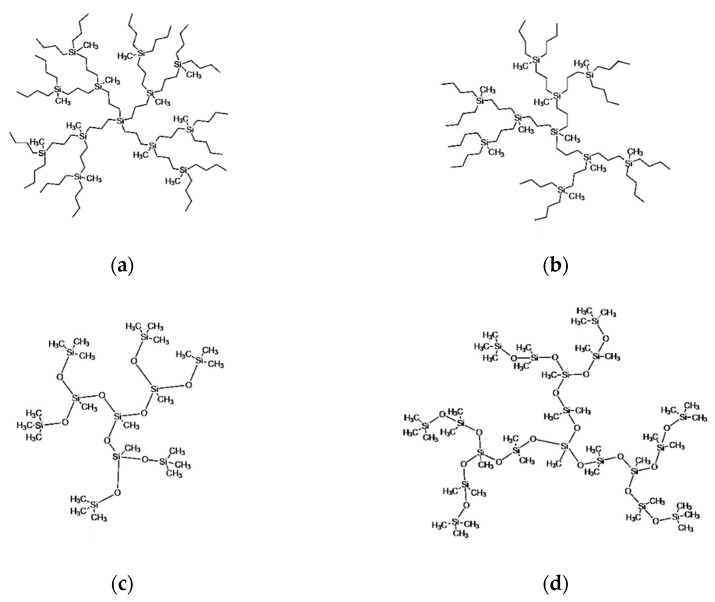
Schematic representation of the second-generation dendrimers belonging to the four homologous series under Scheme C4 dendrimers (**a**), C3 dendrimers (**b**), siloxane S-dendrimers (**c**), and l-dendrimers (**d**).

The adsorption of single dendrimer molecules onto a flat impenetrable solid surface was simulated by the molecular dynamics (MD) method. The silicon-containing dendrimers belonging to four homologous series differing by the architecture and chemical composition of the molecules were considered. The structures of the representatives of each homologue series under study, namely, two types of carbosilane dendrimers and two types of siloxane dendrimers, each of the second generation as an example, are presented in Figure 1. The carbosilane dendrimers shown in the first row differ by the functionality of the core atom, which is equal to four and three in Figure 1a,b, respectively. The siloxane dendrimers in the second row of Figure 1 differ by the length of their spacers. All types of the dendrimers have three-functional ≡Si(CH_3_) branching points, but the siloxane and carbosilane dendrimers have different end segments, namely, methyl -CH_3_ and butyl –(CH_2_)_3_–CH_3_ groups, respectively. The dendrimers from the 4th to the 7th generation are modeled for each homologous series. In the following, the generation of dendrimers is indicated by the symbol i in the designation Gi regardless of the type of dendrimer.

It should be noted that the comparative analysis of the internal structure and segmental mobility of the carbosilane and siloxane dendrimers has been performed earlier in [41]. When discussing the adsorption of these dendrimers, it is convenient to use the dendrimer notations introduced in that work. Namely, siloxane dendrimers with the shorter and longer spacers are denoted as S-dendrimers and L-dendrimers, while the carbosilanes with the three- and four-functional cores are called C3-dendrimers and C4-dendrimers, respectively.

As in Ref. [41], we divide the four series of dendrimers into three pairs for comparison. First, it is a pair of the carbosilane dendrimers that have the same chemical composition, the same spacers, the same branching and end groups but different functionalities of the core atom. Second, these are L- and C3-dendrimers. Despite the different chemical composition, they have rather similar geometric parameters. Indeed, they have almost the same length of spacers, but it is worth considering their different dynamic behavior [41]. Finally, these are siloxane dendrimers, differing in the spacer length.

The general approach to dendrimer modeling was similar to that used in Refs. [41,42]. Molecular dynamics simulations were performed within the PUMA software package [43,44] with the use of the AMBER [45,46] and PCFF [47] force fields for the carbosilane and the siloxane dendrimers, respectively. Methyl and ethylene groups were represented by united atoms. All the simulation details concerning the potentials acting on each atom as well as the preparation of initial non-overlapping dendrimer conformations with correct values of the bond length and valence angles can be found elsewhere [41,48]. Shortly, non-bonded interactions were accounted for by the Lennard–Jones (LJ) potential, Uvw; potentials U12 and U13 describing bond stretching and bond bending, respectively, were introduced for both siloxane and carbosilane dendrimer series in a quadratic form, while the dihedral angle potential, U14, was additionally used for the carbosilane dendrimers. Electrostatic interactions arising from partial charges were calculated using the screened Coulomb potential, Uqq. The validity of the developed MD models for the series of carbosilane and siloxane dendrimers was confirmed by good agreement with experimental data on both dilute solutions and melts [29,49].

A standard MD method with a collisional thermostat [50,51] was used to relax dendrimers at the temperature of 350K. The relaxation of G4 and G5 dendrimers took place for 10 ns, but this equilibration time was enhanced to 20 ns for the G6 and G7 dendrimers according to the results on dendrimer intramolecular dynamics obtained in [41]. To simulate adsorption, an equilibrated dendrimer was placed next to the adsorbing plane at a random orientation. Then, the adsorption potential acting on each dendrimer atoms was applied, and it had the following form:

*U*_ads_ = 0.5*ε*((*R*_min_/*z*)^9^ − 3(*R*_min_/*z*)^3^), (1)

where *z* was the shortest distance between the atom and the adsorbing plane placed at *z* = 0. *R*_min_ was equal to 3 Å, while the energy parameter *ε* was varied from 0 to 1 kcal/mol in increments of 0.2 and from 1 to 3 kcal/mol in increments of 0.5. The cut-off radius was equal to 130 Å, this value being large enough for the entire dendrimer to be affected. This functional form of the adsorption potential corresponds to the standard LJ potential integrated over x and y coordinates in the surface plane. Thus, it implies that the usual van der Waals interactions act between atoms of dendrimer molecules and the adsorbing surface. It should be noted that the parameters of the adsorption potential are the same for all atoms of dendrimers, regardless of their chemical nature.

To study dendrimer adsorption, time trajectories of 20 ns were obtained for all types of G4-G7 dendrimers at every value of the parameter ε. The temperature was fixed at 350 K. The dendrimer characteristics presented in plots below were obtained via time averaging over the last 5 ns and an ensemble averaging over eight independent realizations.

## 3. Results and Discussions

### 3.1. Determination of Allowable ε-Values

**Figure 2:**
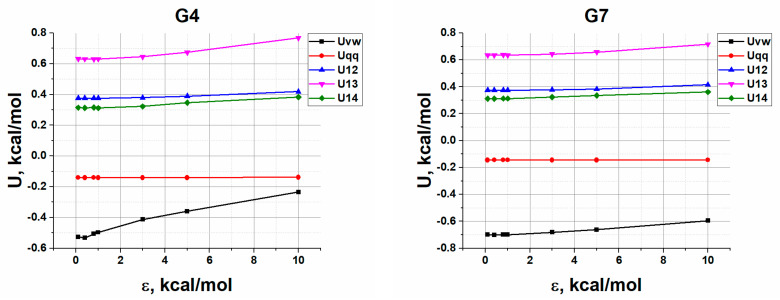
The dependences of the potential energy contributions per atom on the adsorption energy parameter ε calculated for the 4th and the 7th generations of C4-dendrimers. Here, Uvw is the energy of van der Waals interactions, Uqq is Coulomb energy due to partial charges, U12, U13, and U14 are the potential energies of bond, valence angles, and torsional angles, respectively.

Considering, on the one hand, the roughness of the model adsorption potential, but on the other hand, the atomistic proximity of dendrimer models to real systems, it is necessary to determine the values of the adsorption energy ε in Equation (1) at which it makes sense to analyze the results. Obviously, if the adsorption force is too low, dendrimers will not be retained on the surface, or their adsorption will be probabilistic, so the modeling will be time consuming. On the other hand, too strong interactions with the surface can lead to large deformations of bonds and valence angles, which makes modeling using harmonic potentials incorrect. Thus, to determine the feasibility of the model approach, we first calculated the potential energy contributions per atom as functions of the adsorption parameter ε. As an example, Figure 2 shows the corresponding graphs obtained for G4 and G7 carbosilane C4-dendrimers.

One can see that starting from some value of ε, the potential energies of bond and angle deformations start to grow, and so does Uvw. For example, for G4, the increase in U13 for ε = 5 kcal/mol is 7.1%, and for ε = 10 kcal/mol, it is already 21.9%. The change in U-contributions depends on the type and generation of dendrimers; however, our goal is not to accurately determine but simply to estimate the boundaries for ε, where dendrimers are actually adsorbed but still do not experience significant internal stresses. Analysis of the energy contributions shows that considerable bond and angle deformations develop at ε between 3 and 5 kcal/mol; thus, the value of 3 kcal/mol was chosen as the upper limit of ε. This choice seems to be reasonable, since the model is still correct; on the other, one can expect that the regime of a strong adsorption would be reached at this value of the adsorption parameter. For ε = 3 kcal/mol, the change in the energy of all potentials does not exceed 2.5%. A value of 0.4 kcal/mol was chosen as the lower limit for ε so that dendrimers remain adsorbed throughout the time trajectory. The thermal energy of an atom under these conditions is approximately 0.12 kcal/mol, but even with ε = 0.2 kcal/mol, dendrimer adsorption is probabilistic. Thus, the selected ε-range of 0.4–3 kcal/mol describes the model behavior of the dendrimers in both weak and strong adsorption regimes, in which the presence of adsorption and the correctness of the model can be unambiguously asserted.

Nevertheless, it is important to note that we model dendrimer adsorption caused by van der Waals attraction and so high adsorption strength such as 3 kcal/mol could be difficult to achieve experimentally in the absence of any naked charges on the adsorbing surface. For example, the characteristic energies of atomic interaction with various surfaces (carbon, silicon, gold, and some others) take values from 1 to 1.7 kcal/mol [52]. However, the influence of additional factors, such as hydrophobic interaction, surface roughness, and others, which can increase the effective adsorption potential, could be taken into account by enhancing adsorption energy range. We emphasize that the rough model of the adsorbing surface that is used in this work does not make any distinctions in the interaction of atoms of different nature with the substrate. However, it allows focusing on the effects of a particular atomic structure of silicon-containing dendrimers, i.e., the presence of bond, valence angle, and torsion angle potentials, as well as additional atomic groups in spacers and at branching points, on their conformational behavior during adsorption. The study of the influence of different interaction forces acting on atoms of specific types near specific substrates modeled at an atomistic level may be a topic for future research in the context of complicating the model. It is expected that this difference should have a lesser effect than the presence of bond potentials and additional atomic groups in the structure, since usually, the real difference between van der Waals forces acting between atoms of different nature with the same substrate is quite small and amounts to tenths of kcal/mol [52].

### 3.2. Relative Number of Contacts

**Figure 3:**
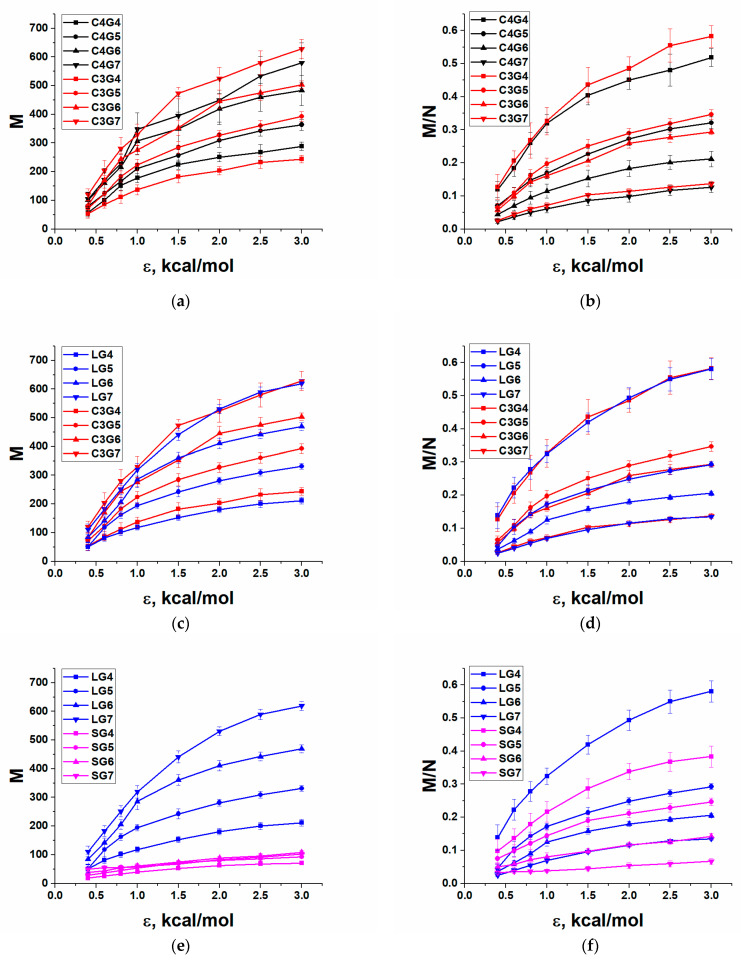
Dependences of the absolute, M, (**a**,**c**,**e**) and relative, M/N, (**b**,**d**,**f**) number of the adsorbed atoms on the adsorption energy parameter ε for the carbosilane and the siloxane dendrimers of various generations. The dendrimer types are indicated in figure legends.

To characterize dendrimer adsorption, we calculated the number of atoms, M, trapped in a thin adsorption layer near the surface as a function of ε. The atom was considered adsorbed if it was at a distance smaller than or equal to 5 Å from the adsorbing plane. Figure 3 shows the absolute, M, and relative, M/N, numbers of the adsorbed atoms, where N is the total number of atoms in the dendrimer. Results are grouped for reasonable comparison.

The general trends that are clearly seen in Figure 3 are as follows. Obviously, the absolute number of adsorbed atoms increases with the dendrimer generation and the strength of adsorption, regardless of the type of dendrimers. However, in relative values, lower-generation dendrimers are more strongly adsorbed, i.e., M/N decreases with dendrimer generation at any ε for all dendrimers under study. Thus, the lower the dendrimer generation, the more substantial conformational transformations dendrimers experience.

Despite these general trends, there are large differences in numbers. Comparing the behavior of dendrimers belonging to different homologous series, one can say that the smallest number of contacts with the surface is realized for the S-dendrimers at any ε. This result is quite expected, since the S-dendrimers have the shortest possible spacer consisting of only one oxygen atom, and the total amount of atoms in these dendrimers in each generation is minimal. However, not only the absolute but also the relative number of adsorbed atoms is minimal for the S-dendrimers. A weaker adsorption of S-dendrimers is especially pronounced in the region of large ε-values. A short spacer seems to be the main obstacle to conformational changes. Thus, the G7 S-dendrimer with the densest molecular structure that is somewhat stressed even in a non-adsorbed state [53] practically does not change its conformation, regardless of the adsorption strength in the chosen range of ε. The relative number of contacts only slightly exceeds 7% at the largest ε = 3 kcal/mol. It should be noted that within the coarse-grained simulations realized in Ref. [25], it has been shown that for the G7 dendrimer with the shortest spacer, the fraction of monomers in contact with the surface saturates at about 0.6, which reflects packing constraints in 2D. Within the atomistic approach accounting for realistic values of the adsorption energy, this limiting case is hardly accessible.

On the other hand, the siloxane L-dendrimers, as well as the carbosilane C3 and C4 dendrimers, have longer spacers of almost the same length and, thus, acquire a greater capacity for conformational transformations during adsorption. The largest number of adsorbed atoms is realized for C3 dendrimers. This is quite surprising, since they contain fewer atoms than C4 dendrimers with an extra dendron coming from the core Si atom. Only the fourth generation of C4 dendrimers surpasses the analogous generation of C3 dendrimers. In addition, C3 and L-dendrimers of the seventh generation have almost the same values of M at any ε.

As for the relative number of contacts, it can be seen that for the fourth generation there are practically no differences between L-, C3 and C4 dendrimers, only at high ε-values does a slightly poorer adsorption of C4 dendrimers become noticeable. That is, for “small” generations, the chemical nature of the dendrimers together with some differences in the segmental mobility between carbosilane and siloxane dendrimers do not play any role, and the geometric parameters are decisive in accordance with theoretical predictions. Comparing the fifth generations, one can see that both carbosilane dendrimers adsorb better than the siloxane ones. This means that the presence of the fourth dendron in C4 has less effect than the nature of dendrimer spacers. For the sixth generation, there are no fundamental differences in adsorption between C4 and L-dendrimers. This means that the methyl groups in the middle of the spacers of the L-dendrimers create similar steric restrictions for the change in conformation upon adsorption, as does the fourth dendron of C4 dendrimers. However, C3 dendrimers, which do not have such obstacles, are adsorbed better: they are practically indistinguishable from the fifth generation of C4 and L-dendrimers. Finally, the adsorption behavior of G7 dendrimers is practically independent of the dendrimer type. That is, starting from a certain generation, the influence of the generation itself overlaps the effects due to the structure of dendrimers as soon as they have the same spacer length.

Comparing the adsorption behavior of L-, C3, and C4 dendrimers, one can conclude that at a given spacer length, it ceases to depend on the chemical nature of dendrimers starting from the seventh generation. However, in the region of intermediate generations, when, on the one hand, structural features of dendrimers start to play a role, and on the other hand, the dense globular conformation has no decisive influence yet, dendrimer behavior is affected by such factors as the specific types of bonds and angles, their mobility, as well as the presence of additional atoms in the structure, which cannot be ignored.

Thus, for all types of dendrimers, from the point of view of adsorption, conditionally “low” generations can be distinguished; this is the fourth one for C4, C3, and L-dendrimers, for which the adsorption strength depends mainly on geometric parameters. “Medium” generations are the fifth and the sixth for C4, C3, and L-dendrimers and the fourth and the fifth for S-dendrimers. In this range of generations, dendrimer adsorption depends not only on geometric parameters of macromolecules but also on differences in dendrimer internal structure and composition. Finally, “high” generations can be distinguished when the influence of a dense molecular structure starts to dominate and erases all other differences between the types of dendrimers. These are the sixth generation for S-dendrimers and the seventh one for the other dendrimers. Separately, the seventh generation of S-dendrimers can be viewed as a very rigid structure, which practically does not experience any conformational transition.

It is also worth noting that these differences are clearly visible at high adsorption strengths when considerable conformational changes seem to take place. At low ε-values, in the weak adsorption regime, the dependences lie closer to each other, and it is much more difficult to highlight on their basis any differences in the adsorption behavior of dendrimers depending on their chemical composition and architecture. Drawing an analogy with the data available in the literature and those mentioned above, it can be assumed that dendrimers will behave similar in relative units when adsorbed in low concentrations on smooth glass, and they are quite distinguishable when adsorbed on gold at sufficiently high concentrations.

### 3.3. Number of Atoms in Contacts with Surface per Structural Layer

The adsorption of dendrimers undoubtedly causes some changes in dendrimer conformations. Similar to other macromolecules, dendrimers are expected to spread out on the surface to increase the number of contacts with the surface and to enhance the energy gain upon adsorption. However, in contrast to linear polymers, dendrimers have greater limitations in the degree of conformational changes due to their tree-like architecture. It has been shown [25] that under a weak adsorption, only peripheral atoms interact with the substrate, while under strong adsorption, dendrimers could structurally undergo the transition from a nearly spherical to a pancake conformation so that the atoms belonging to the internal region of a dendrimer molecule could also interact with the surface.

**Figure 4:**
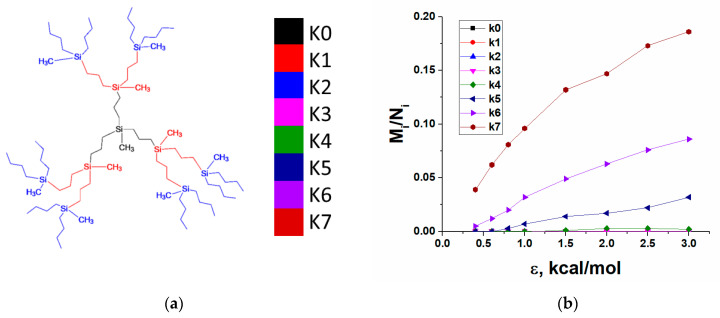
(**a**) Schematic representation of the classification of structural layers. Different colors show different layers numbered from the dendrimer core, Ki corresponds to the layer number. (**b**) Dependence of the number of contacts in each structural layer on the adsorption energy parameter ε calculated for the seventh generation of the C4 dendrimer.

To analyze in detail which dendrimer atoms are exposed to the adsorbing surface depending on dendrimer generation and chemical composition, we calculated the relative number of contacts with the surface, which are formed by atoms belonging to different structural layers of dendrimers as a function of the adsorption energy ε. By a structural layer, we mean a group of Si-branching atoms characterized by the same topological distance from the dendrimer core and also all atoms of the spacers outgoing from these branching points. This division of a dendrimer into structural layers is illustrated by the example of the second generation C3 dendrimers in Figure 4a. For other generations, the classification is similar. It is also worth noting that the number of atoms in each subsequent layer is at least doubled, so one should be careful when comparing the layers with each other. In addition, one should take into account the difference in the end groups of siloxane and carbosilane dendrimers; however, it does not affect the main qualitative conclusions that can be drawn from this analysis.

**Figure 5:**
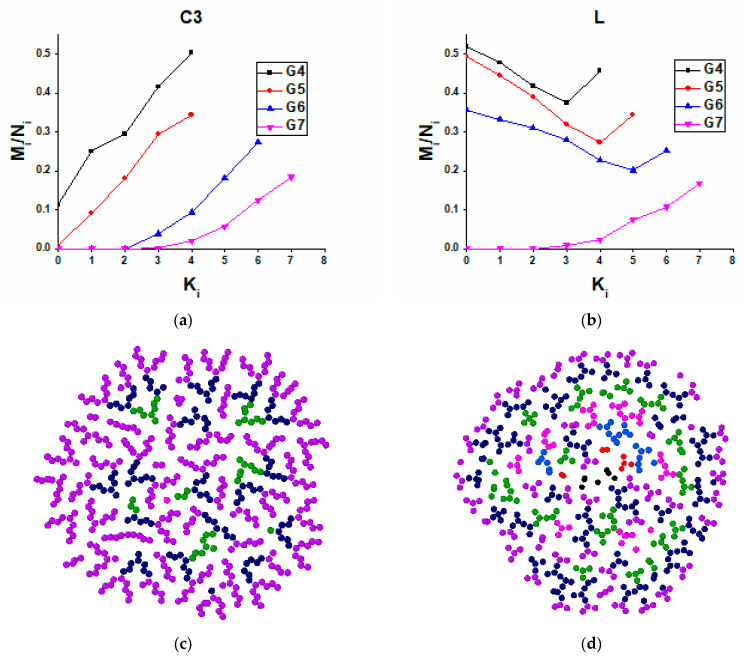
Dependences of the relative number of contacts on the number of the structural layer at ε = 1.5 kcal/mol for (**a**) C4 and (**b**) l-dendrimers. Frontal projections of the adsorption layer of 5 Å for (**c**) C3 and (**d**) L-dendrimers of the sixth generation. Different colors are used for atoms belonging to different structural layers, color palette vs. ki, which are shown in Figure 4a.

As expected, G4 dendrimers with the least dense structure can realize contacts with the surface within all structural layers (Figure S1) while higher-generation dendrimers behave differently. As an example, Figure 4b shows the relative number of contacts realized by atoms of each structural layer as a function of the adsorption energy for the G7 C4 dendrimer. One can see that only three peripheral layers have noticeable numbers of contacts with the surface even at the maximal ε, while a few inner layers are not exposed to the surface at all. This behavior is typical for all higher generations of C4, C3, S-, and the seventh generation of the L-dendrimer; i.e., these molecules interact with the surface practically without violating the integrity of their internal structure. At a fixed ε, the relative number of contacts within each structural layer monotonically increases with the layer number, while the unperturbed inner part of dendrimers grows with generation (see plots presented in Figure 5a for C3 dendrimers).

A striking result was found for L-dendrimers below the seventh generation. In contrast to the other dendrimers, they adsorb preferentially by atoms belonging to internal layers (Figure 5b). The qualitative difference in the conformational behavior of these dendrimers during adsorption is also clearly seen from a comparison of the frontal projections of adsorption layers, for example, of the sixth generation of C3 and L-dendrimers shown in Figure 5c,d. The frontal projections obtained for the other dendrimers can be found in the Supplementary Material (SM).

It can be expected that the key factor in the qualitative difference between the mechanisms of adsorption of L-dendrimers and dendrimers of the carbosilane series is associated with the presence of more flexible Si-O-Si bonds as compared to CH_2_-CH_2_-CH_2_ in the latter. However, the molecular structure of dendrimers becomes denser with an increasing generation number, and it becomes the leading factor governing the adsorption behavior of G7 L-dendrimers. It is the dense molecular structure that seems to also play a leading role in the adsorption of S-dendrimers with the shortest spacers neutralizing the influence of the flexible Si-O-Si bonds in these dendrimers.

Summarizing, we can say that the peripheral layers of the two carbosilane dendrimers, S-dendrimers, as well as the seventh generation of L-dendrimers, act as a kind of a shell during dendrimer adsorption. That is, these dendrimers are adsorbed in a manner similar to a rubber ball filled with liquid, which changes its shape without any violation of the integrity of its surface. On the contrary, the L-dendrimers of the other generations spread out over a plane, so that atoms in the interior interact to a large extent with the surface. From the point of view of the total number of contacts, geometrically identical C3 dendrimers are adsorbed even better at the same generation. In addition, recall that all atoms are adsorbed with the same potential, and such difference in behavior is solely due to the structural features of specific dendrimers. This finding should be important for practical applications of silicone-containing dendrimers, in the case of any special modifications to the external or internal parts of dendrimer molecules.

### 3.4. Density Profiles and Shape Changes

The adsorption of almost spherical dendrimer molecules to a flat surface obviously violates spherical symmetry. Thus, to characterize conformational changes during adsorption, we calculated two kinds of density profiles, namely, the profiles perpendicular and parallel to the adsorbing plane. The former one was defined as the linear density in z-direction:

ρ_⊥_(*H*) = ∑*n*_i_(*H*)*m*_i_/d*H* (2)

where *n*_i_ is the number of atoms of the sort i in a flat layer of the width d*H* parallel to the surface and located at a distance *H* from it, *m*_i_ is the mass of i atoms, and d*H* = 0.2 Å. In fact, this profile shows how the density changes with distance from the plane along the z-axis oriented perpendicular to the surface. The parallel density profile was calculated by the formula

ρ _∥_(*R*) = ∑*n*_i_(*R*)*m*_i_/*S*_i_, (3)

where *R* is the distance along the xy plane from the center of mass of the dendrimer to the corresponding annular layer, and *S*_i_ is the area of this layer. Thus, this profile shows how the density changes with distance from the center of mass parallel to the plane.

**Figure 6:**
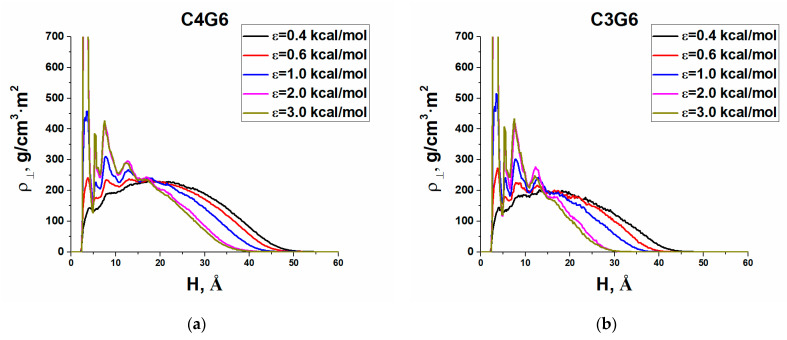

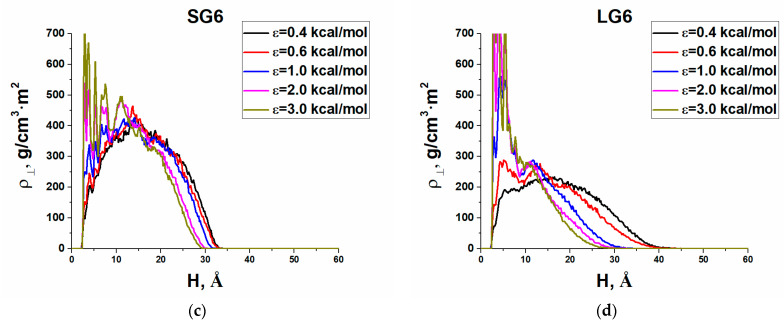
Density profiles ρ_⊥_ perpendicular to the surface for the sixth generation of (**a**) the C4 dendrimer, (**b**) the C3 dendrimer, (**c**) the S-dendrimer, and (**d**) the L-dendrimer.

**Figure 7:**
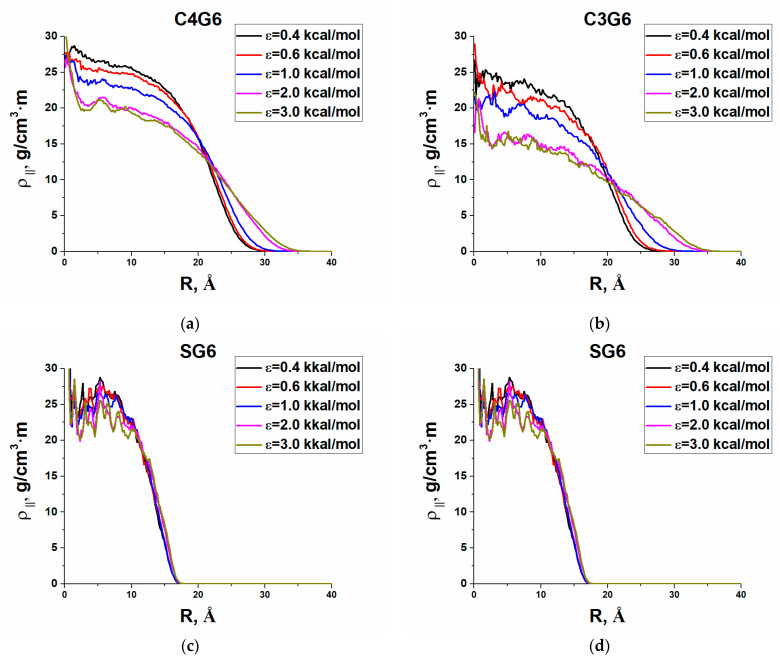
Density profiles ρ _∥_(**R**) parallel to the surface for the sixth generation of (**a**) the C4 dendrimer, (**b**) the C3 dendrimer, (**c**) the S-dendrimer, and (**d**) the l-dendrimer.

Actually, the density profiles reflect a side view of the adsorbing dendrimer and its view from above. Qualitatively, they turned out to be similar for all types of dendrimers. As an example, Figure 6 and Figure 7 show the densities ρ_⊥_(*H*) and ρ _∥_(*R*) obtained for G6 of all dendrimers under study at various values of the adsorption parameter ε. Based on them, one can conclude that at low adsorption energy of 0.4kcal/mol, dendrimers mainly stick to the surface by their peripheral atoms, hardly changing their shape. With an increase in the adsorption energy, a kind of a three-layered structure is formed perpendicular to the plane consisting of (i) a thin strongly adsorbed layer, (ii) a plateau on which heavier silicon layers stand out, and (ii) a falling periphery. Of particular note is only the G7 S-dendrimers (see SM), which are very poorly adsorbed even at the maximal ε. All dendrimers have a disk-like conformation parallel to the plane, with a density peak in the center, which becomes larger and wider with decreasing the generation number. With increasing adsorption energy, the dendrimer dimensions, parallel to the plane, increase except for those of the S-dendrimers that nearly keep their shape. This behavior has qualitatively been predicted by simple models [14]. Furthermore, silicon-containing dendrimers follow the general trends in shape changes predicted theoretically [25] and observed within coarse-grained computer simulations [24,25]. With an increase in the adsorption strength, the dendrimer shape changes less with an increase in generation, an increase in core functionality, or a reduction in spacer length.

**Figure 8:**
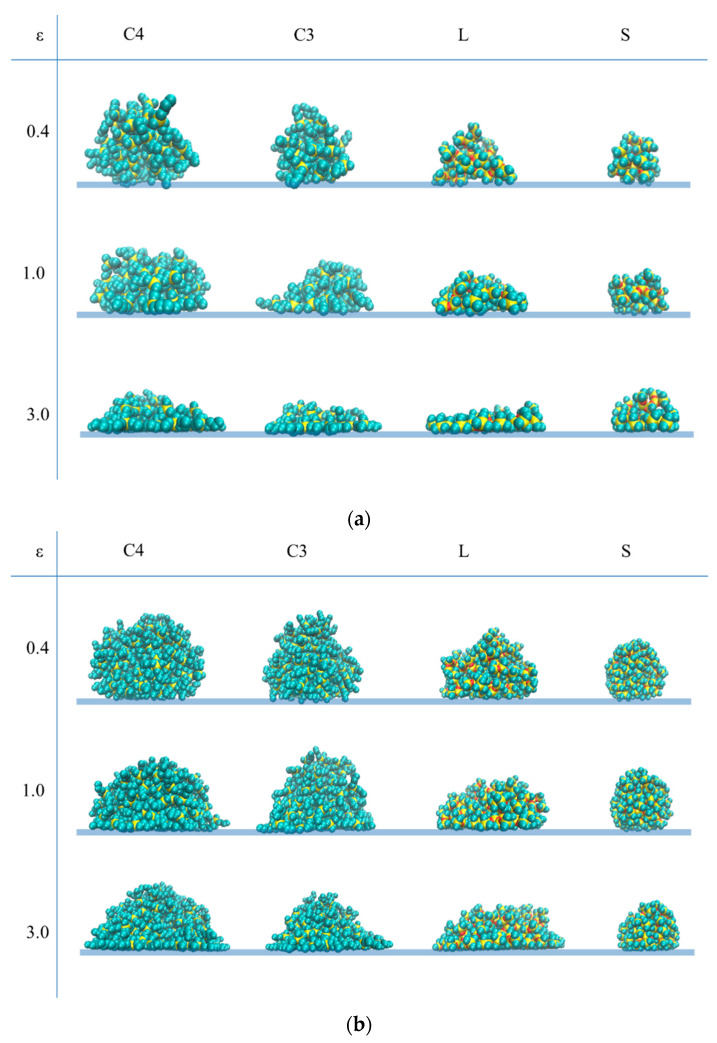
Snapshots of dendrimer conformations (side view) at various values of the adsorption parameter ε for (**a**) the fourth generation and (**b**) the sixth generation of the carbosilane and siloxane dendrimers under study. The yellow beads correspond to Si atoms, red beads correspond to O atoms, and cyan beads show united C atoms.

However, there are some differences in dendrimer conformations depending on the dendrimer type. Together with the general trends, they can also be seen on the snapshots of dendrimer conformations presented in Figure 8 for G4 and G6 generations (snapshots of the other generations can be found in SM). In particular, adsorbed carbosilane dendrimers demonstrate mainly a kind of a “brimmed hat” structure, while siloxane dendrimers spread out in a manner similar to a liquid drop, this picture is in accordance with the results on the structural layer adsorption discussed above.

## 4. Conclusions

In this work, full-atomic molecular dynamics simulations of the adsorption of silicon-containing dendrimers onto a flat surface were carried out. The adsorption of dendrimer atoms was modeled via an attractive potential of LJ (it is defined above) type, mimicking van der Waals interactions with a variable energy parameter to cover both regimes of weak and strong adsorption. Four different homologous series were studied, namely, two types of carbosilane dendrimers differing by the functionality of the core atom and two types of siloxane dendrimers with different lengths of the spacers. Comparative analysis of their adsorption behavior allowed one to elucidate some general trends as well as the influence of dendrimer specificity, namely, the dendrimer generation, the core functionality, spacers length, and the chemical composition.

Among general trends, one should mention an increasing degree of adsorption with decreasing generation number and increasing ε. Despite these general trends, which are similar for all types of dendrimers and coincide with those predicted by theoretical models and coarse-grained simulations, the exact boundaries between weakly adsorbed and strongly adsorbed states strongly depend on the specific structure of dendrimers.

It was shown that the adsorption scenario of siloxane dendrimers with the shortest spacer consisting of only one oxygen atom is different from that of the other dendrimers. A very tight molecular structure of this dendrimer prevents it from considerable deformations during adsorption in the studied range of adsorption energies when dendrimer interactions with the surface do not cause any significant stress in bonds and valence angles. Thus, this dendrimer has a minimal amount of both absolute and relative numbers of contacts with the surface than the other dendrimers for any generation number and any ε.

Comparison of the adsorption behavior of dendrimers with almost the same length of the spacers, namely, carbosilane C3 and C4 dendrimers and siloxane L-dendrimers showed that the fraction of adsorbed atoms of these dendrimers depends on the dendrimer type for “intermediate” G5 and G6 dendrimers but becomes almost the same for G7 dendrimers when the tight structure of this high generation plays a major role in conformational changes during adsorption. G4 realizes the largest number of contacts with the surface; it decreases with generation at a particular value of ε.

A striking difference in the conformational behavior of the carbosilane and the L-dendrimers was found. While the carbosilane dendrimers interact with the surface mainly by atoms of the peripheral structural layers, that is, located at a large topological distance from the **Si** core, L-dendrimers up to the G6 interact preferably by the atoms belonging to inner layers of dendrimer molecules twisting their outer branches outward from the surface. This behavior is manifested in the density profiles perpendicular to the plane, namely, G4–G6 L-dendrimers spread over the plane adopting a pancake shape at large ε, in contrast to the other types of dendrimers keeping its internal part unperturbed and thus adsorbing via “brimmed hat” conformations. Furthermore, a comparison of various generations of C3 and C4 carbosilane dendrimers showed that C3 dendrimers adsorb better in relative units. At a given generation number, C3 dendrimers have a smaller unperturbed inner part than C4 dendrimers, which is presumably due to the steric restrictions caused by the presence of an additional dendron in the latter. The unperturbed inner part grows with dendrimer generation at a fixed ε.

The revealed features of the adsorption of dendrimers, depending on their chemical composition and architecture, provide a new understanding of the general effect of bond flexibility and are very important for the development of new silicon-containing dendrimer structures and materials based on them.

## Supplementary Materials

The following are available online at https://www.mdpi.com/2073-4360/13/4/552/s1, Figure S1: Dependences of the relative number of contacts on the number of the structural layer at ε = 1.5 kcal/mol, Figure S2: Adsorption spot snapshots, ε = 3, C4 dendrimers, Figure S3: Adsorption spot snapshots, ε = 3, C3 dendrimers, Figure S4: Adsorption spot snapshots, ε = 3, L-dendrimers, Figure S5: Adsorption spot snapshots, ε = 3, S-dendrimers, Figure S6: Snap shots for different generations and ε values for C4 dendrimers, Figure S7: Snapshots for different generations and ε values for C3 dendrimers, Figure S8: Snapshots for different generations and ε values for L-dendrimers, Figure S9: Snapshots for different generations and ε values for S-dendrimers.
