# Supplementary material for: Adsorption of Silicon-Containing Dendrimers: Effects of Chemical Composition, Structure, and Generation Number
Source: Polymers (Basel). 2021 Feb 13;13(4):552. doi: 10.3390/polym13040552 (PMC7917902; doi:10.3390/polym13040552)
Supplement: Supplementary file 1 [file polymers-13-00552-s001.pdf]

Supplementary Materials

Figure S1. Dependences of the relative number of contacts on the number of the structural layer at ε=1.5 kkal/mol

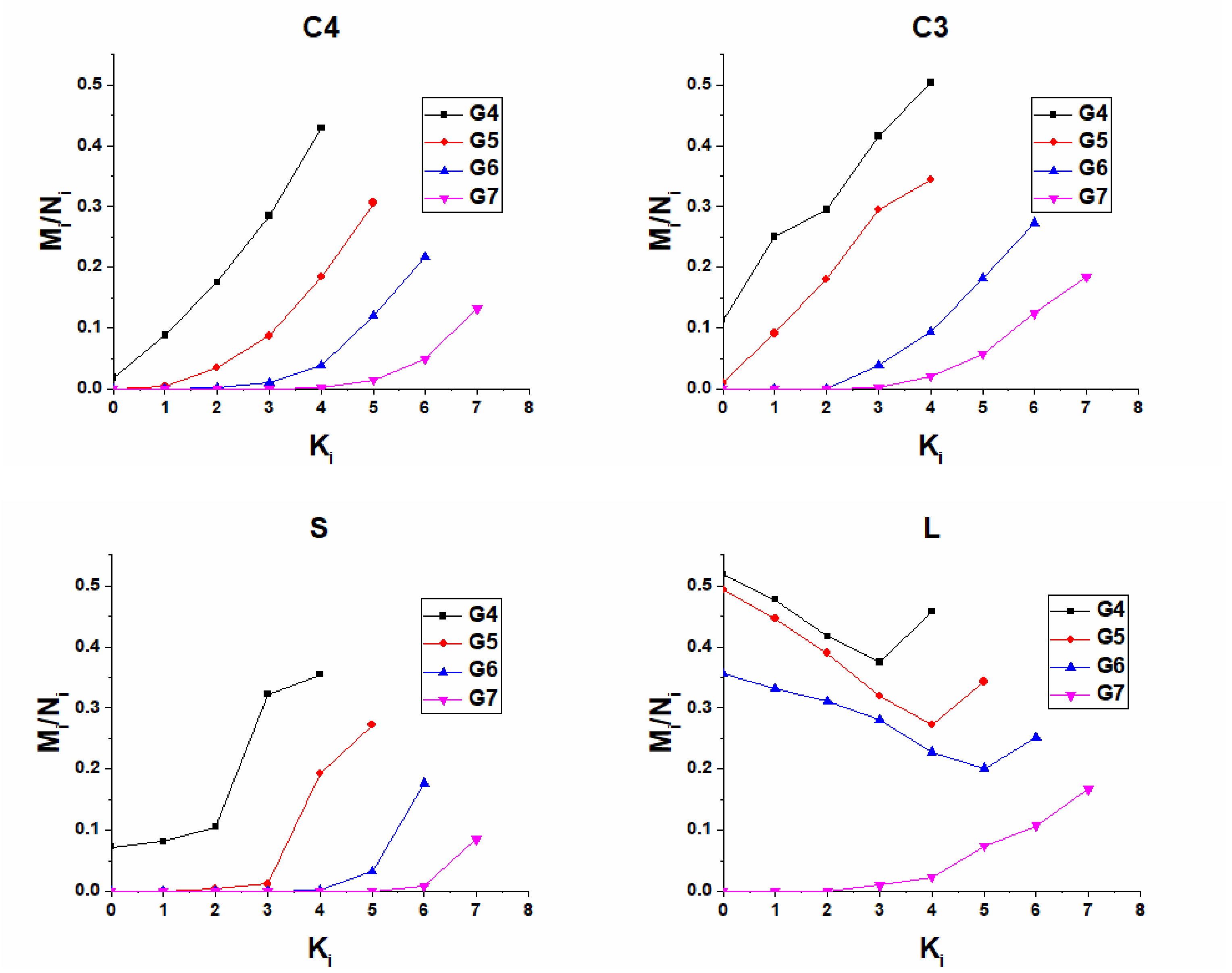

Figure S2. Adsorption spot snapshots, ε=3, C4-dendrimers

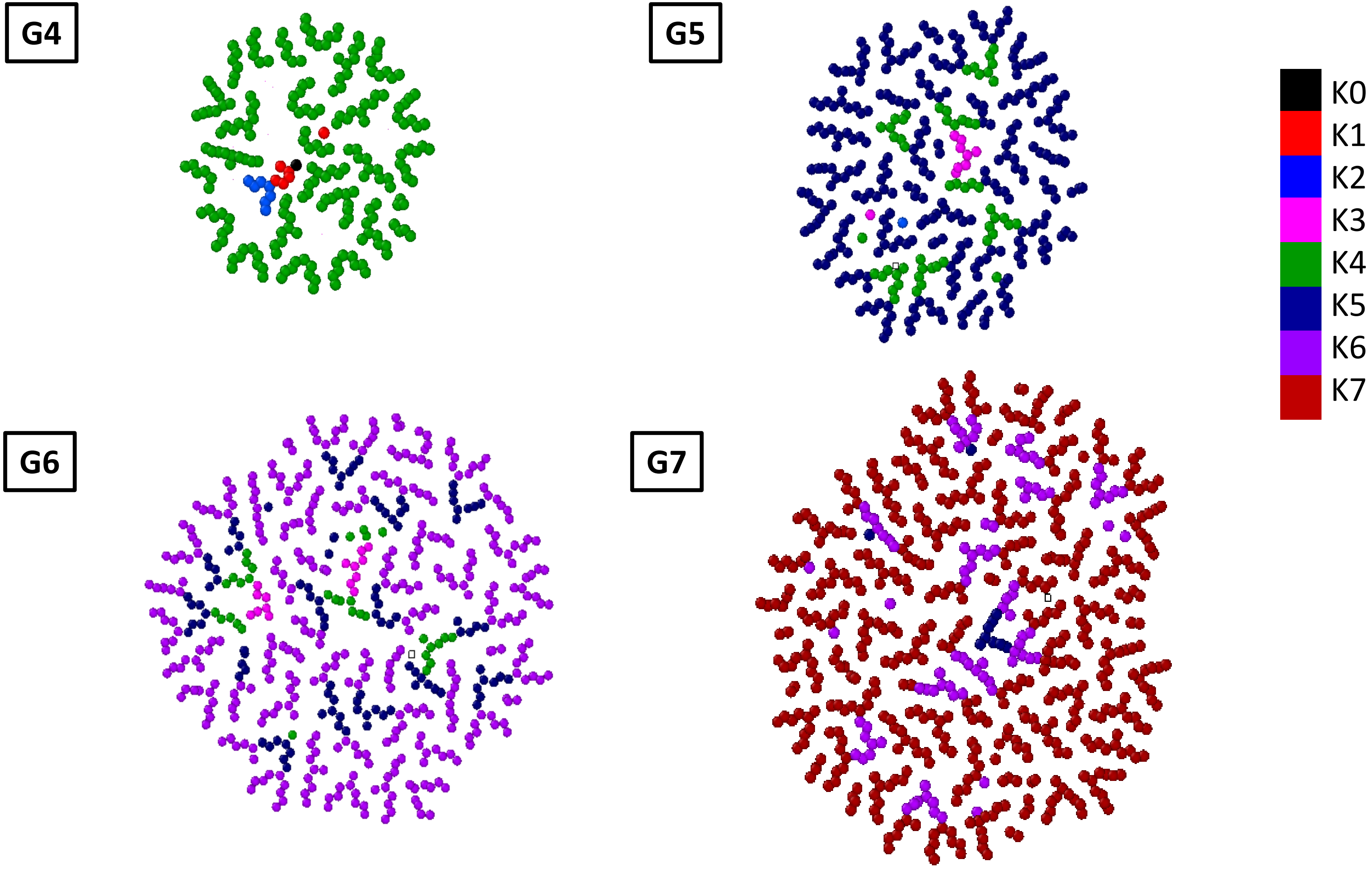

Figure S3. Adsorption spot snapshots, ε=3, C3-dendrimers

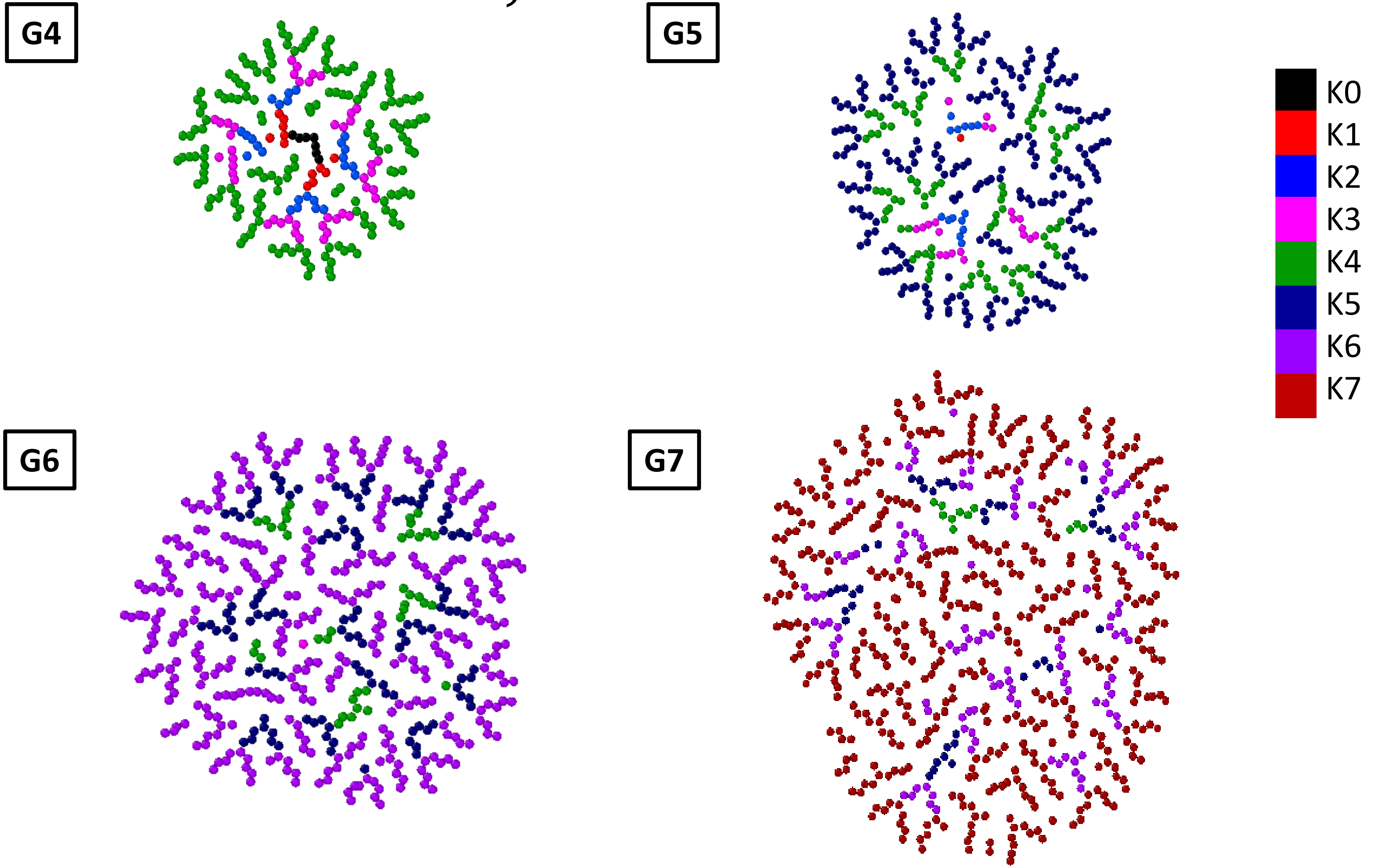

Figure S4. Adsorption spot snapshots, ε=3, L-dendrimers

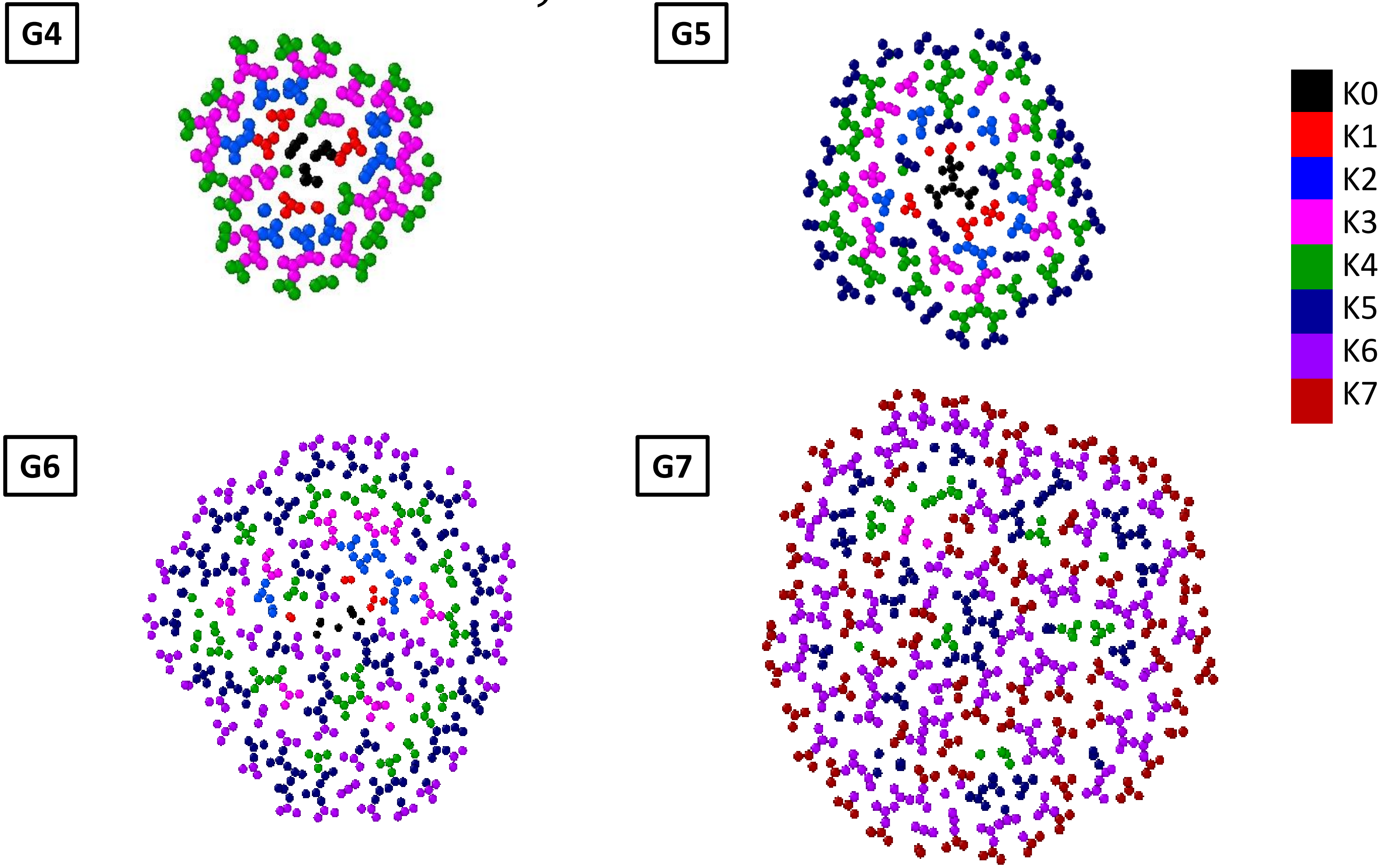

Figure S5. Adsorption spot snapshots, ε=3, S-dendrimers

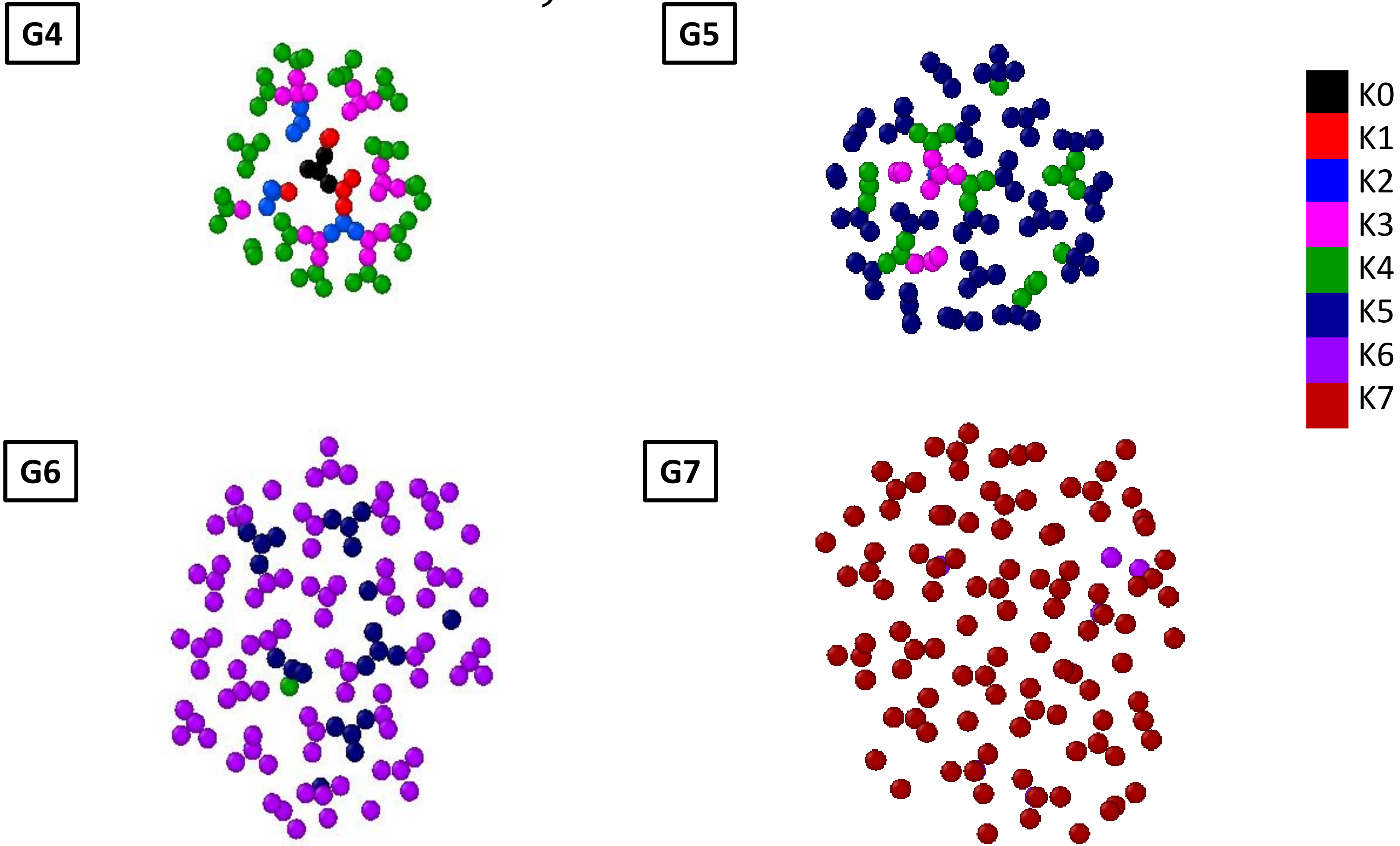

Figure S6. Snapshots for different generations and ε values for C4-dendrimers*

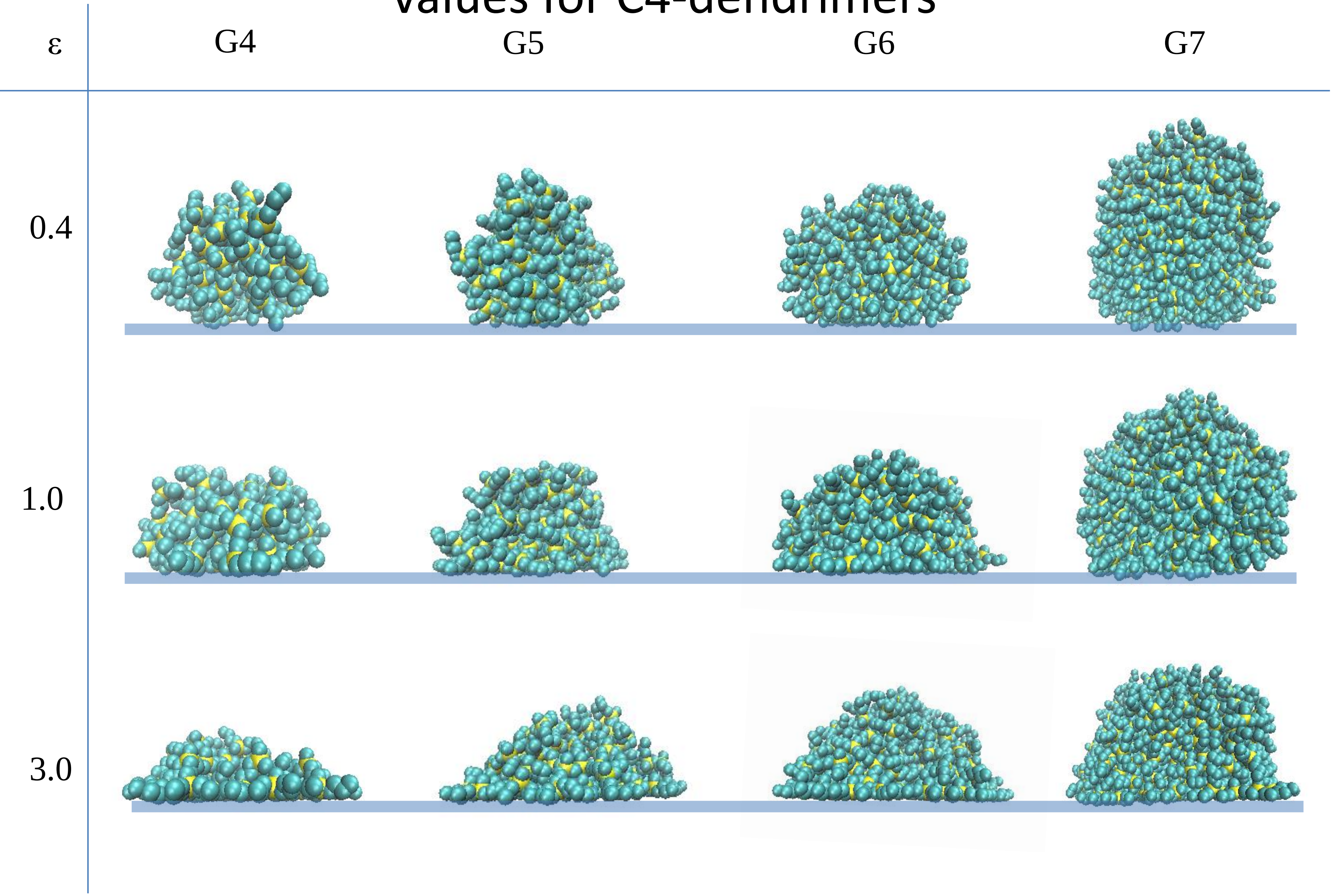

*The yellow beads correspond to Si-atoms and cyan beads to united C-atoms.

Figure S7. Snapshots for different generations and ε values for C3-dendrimers*

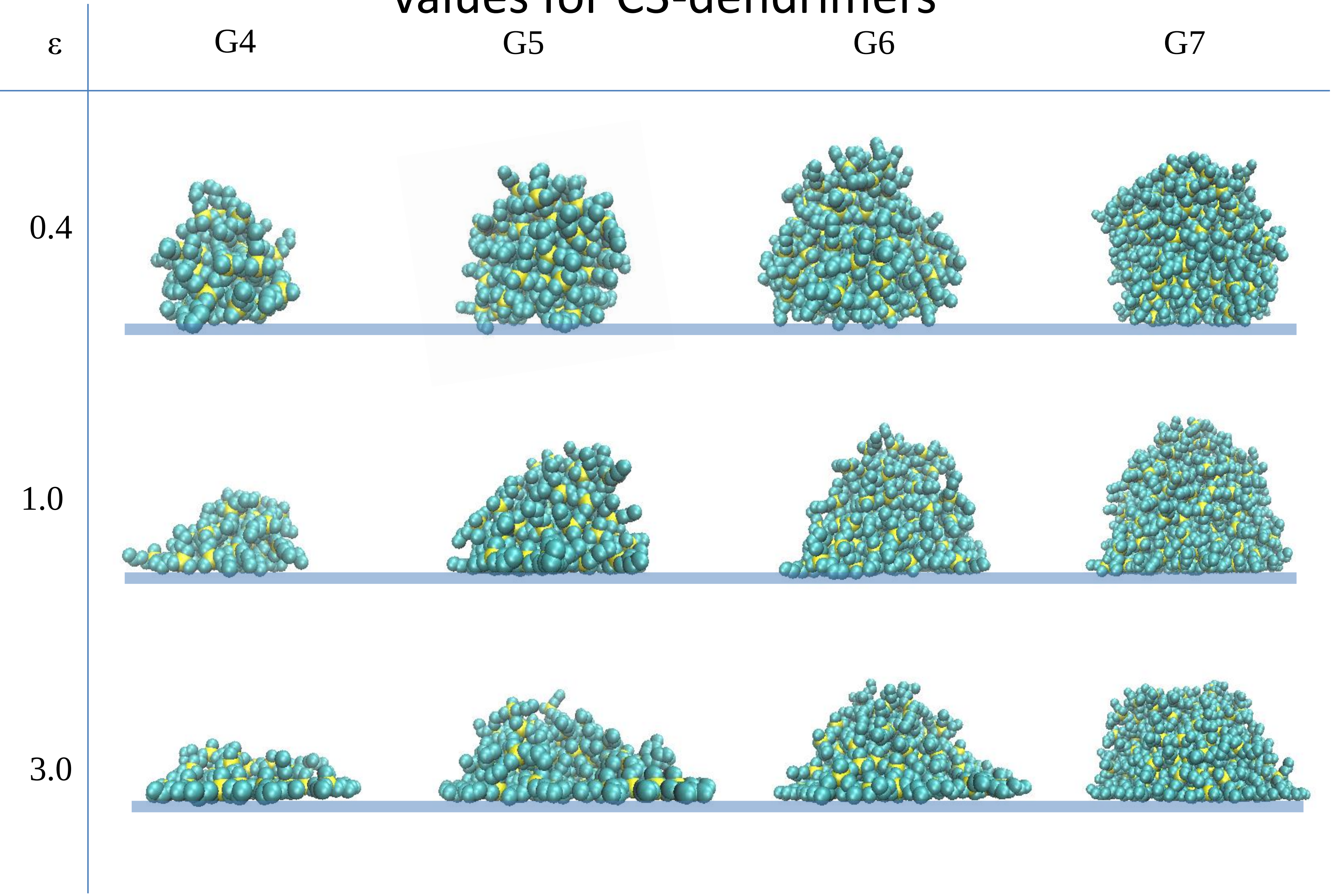

*The yellow beads correspond to Si-atoms and cyan beads to united C-atoms.

Figure S8. Snapshots for different generations and ε values for L-dendrimers*

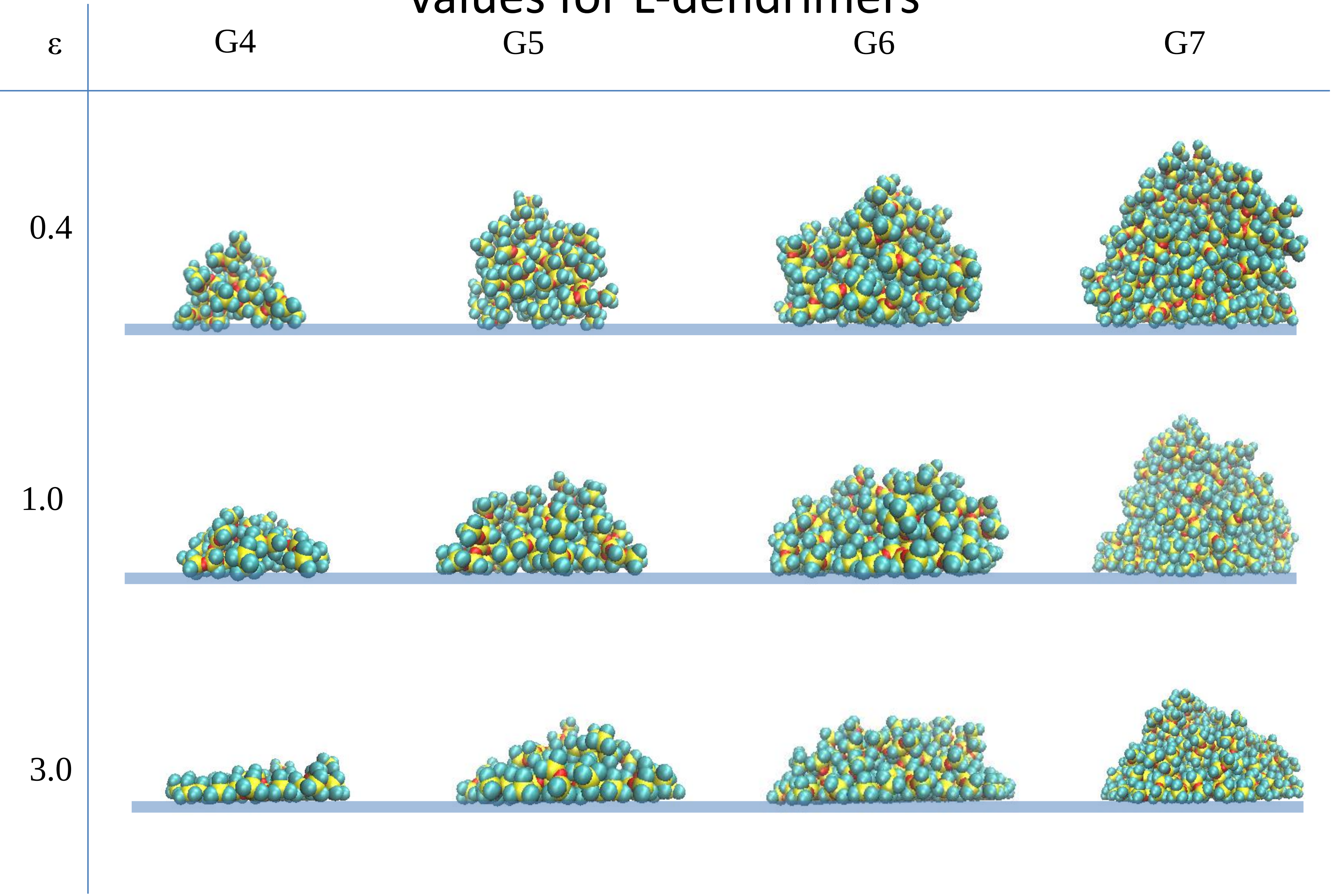

*The yellow beads correspond to Si-atoms, red beads correspond to O-atoms and cyan beads to united C-atoms.

Figure S9. Snapshots for different generations and ε values for S-dendrimers*

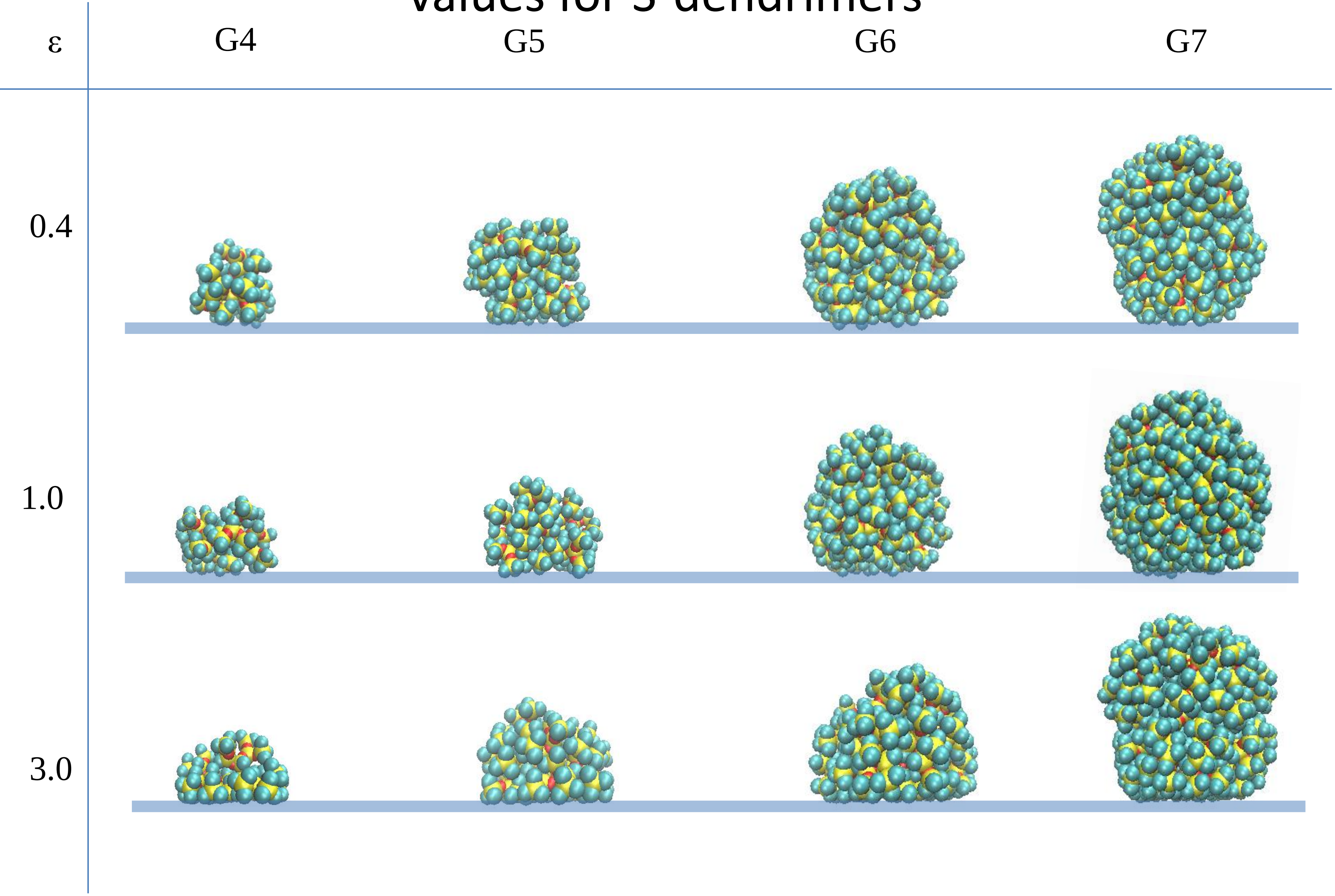

*The yellow beads correspond to Si-atoms, red beads correspond to O-atoms and cyan beads to united C-atoms.
